# Supplementary figures and images for: Species recovery and recolonization of past habitats: lessons for science and conservation from sea otters in estuaries
Source: PeerJ. 2019 Dec 10;7:e8100. doi: 10.7717/peerj.8100 (PMC6910117; doi:10.7717/peerj.8100)

Elkhorn Slough, Surveys and Estimated Trend

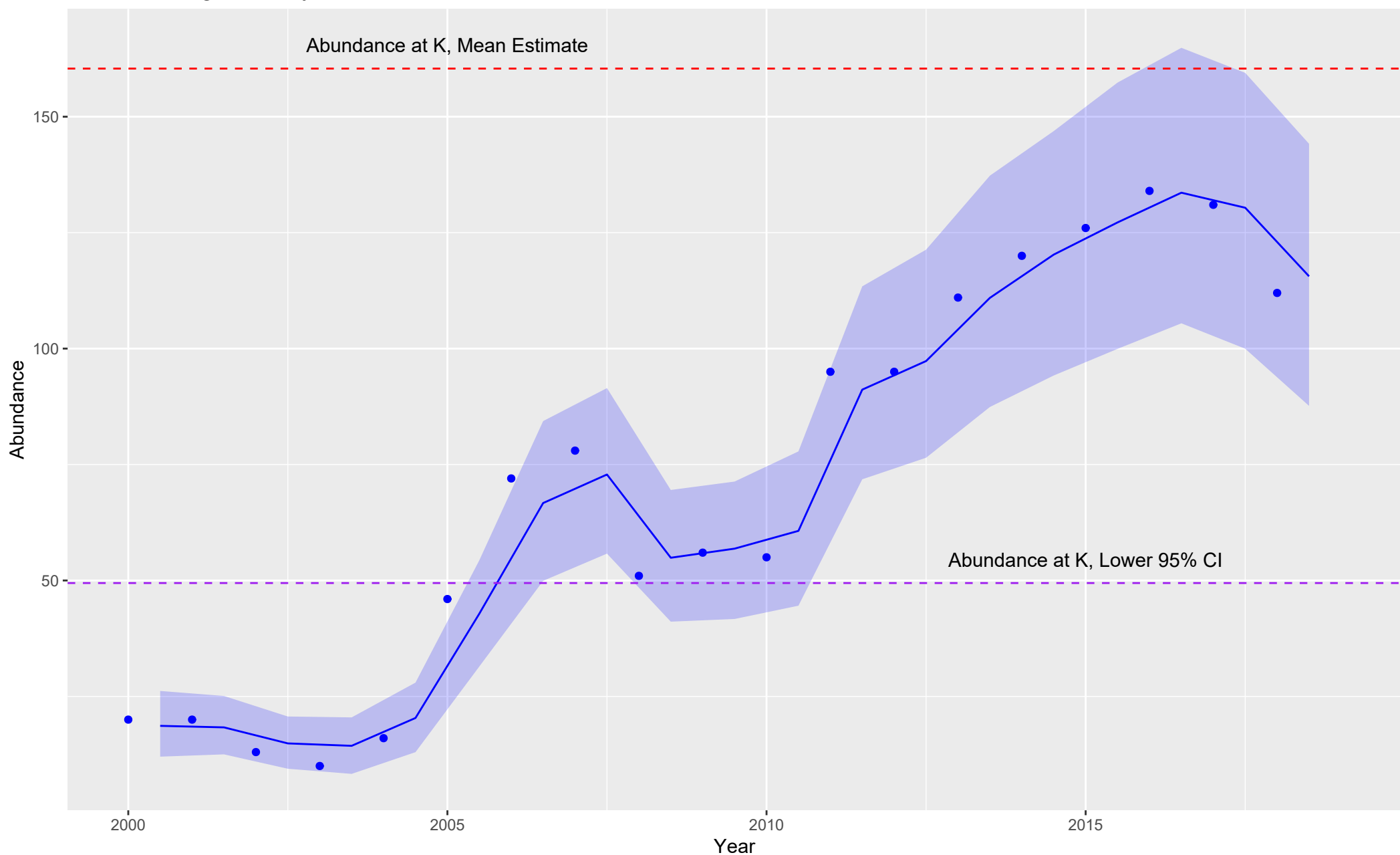

Supplement: Supplemental Information 6 — Plot of survey data of adult sea otter abundance from Elkhorn Slough, 2000–2018 (points), with fitted state space model showing estimated true abundance over time (solid line) and associated 95% CI of the estimated trend (shaded band). Dashed lines show estimated values of K, both the mean estimate (red dashed line) and the lower 95% CI of the K estimate (purple dashed line); the latter value was used to parameterize simulations for San Francisco Bay. [file peerj-07-8100-s006.pdf]

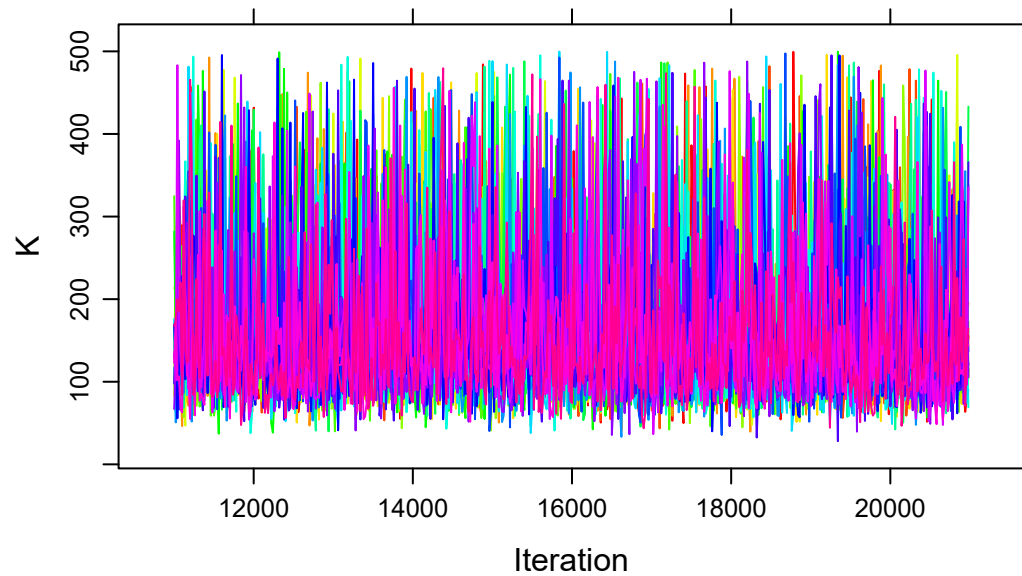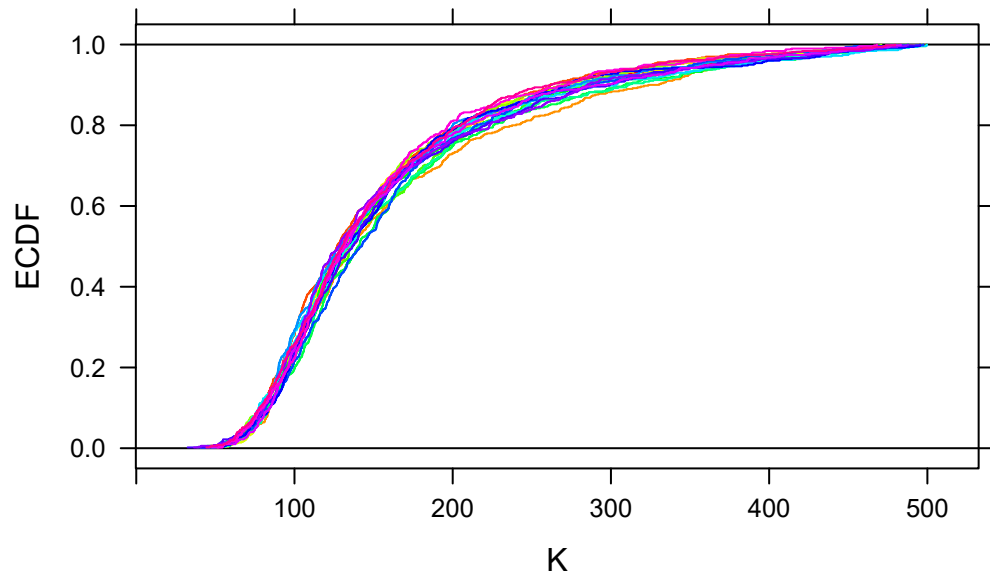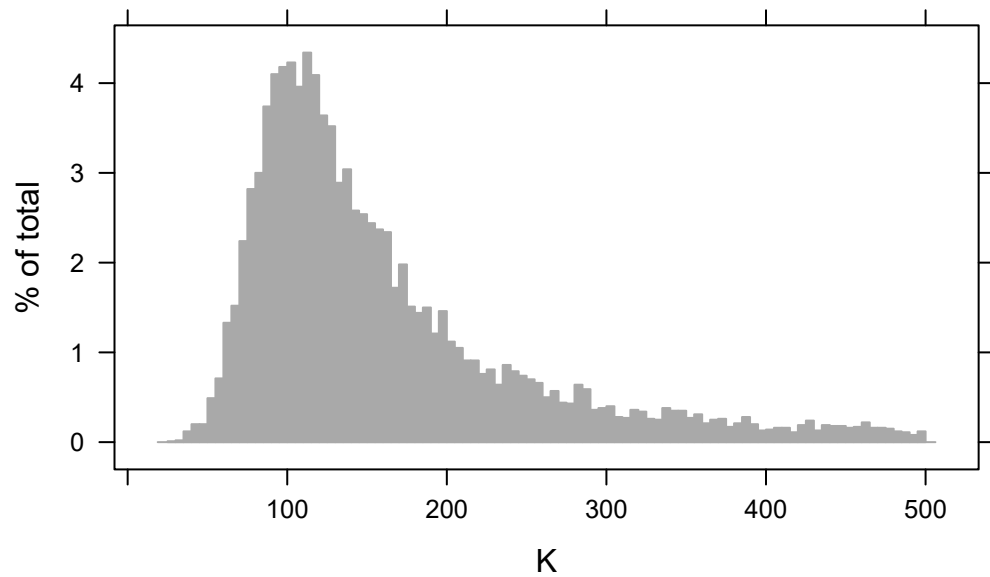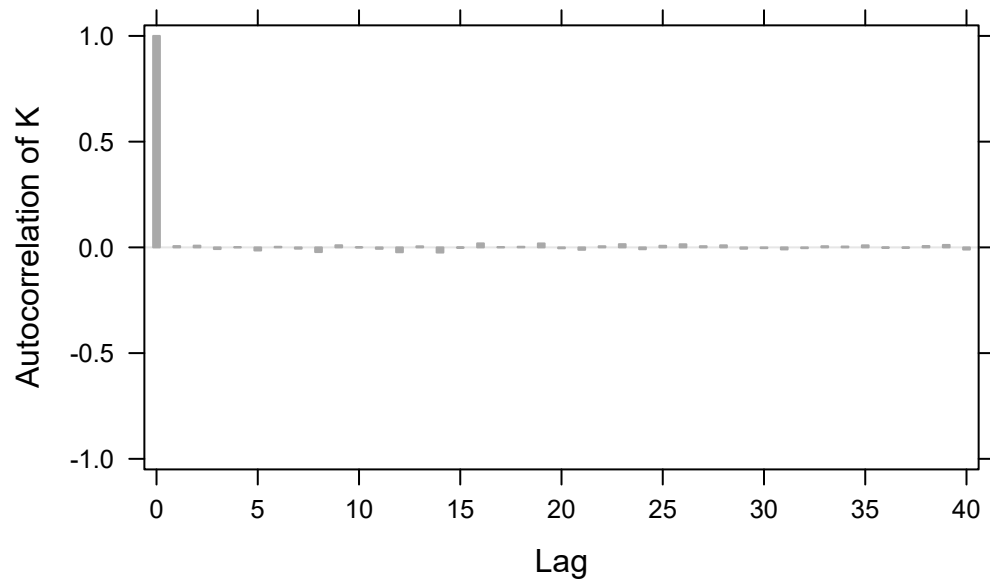

Supplement: Supplemental Information 7 — Diagnostic plots based on posterior samples for parameter “K,” estimated by the state-space model. Top left: Coda Trace plot for 20 chains; Top right: empirical cumulative distribution function (ECFD) for 20 chains; Bottom left: density distribution for posterior samples from 20 chains; Bottom right: lag-k autocorrelation plot. Showing the correlation between each posterior sample and the sample k steps before. [file peerj-07-8100-s007.pdf]

# Posterior predictive check for sum of squared Pearson residuals

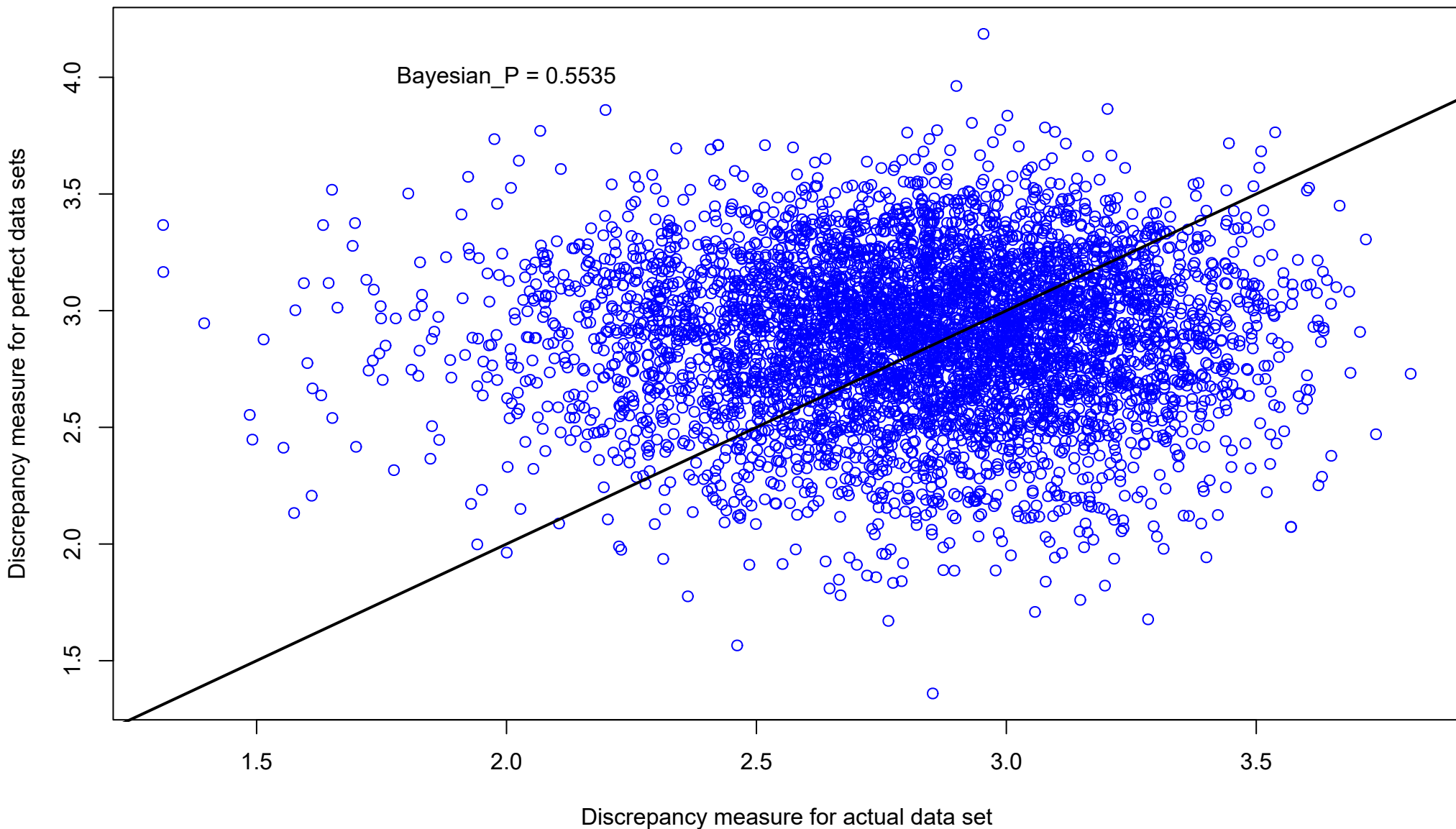

Supplement: Supplemental Information 8 — Diagnostic plots based on posterior samples for parameter “sp” (process error) estimated by the state-space model. Top left: Coda Trace plot for 20 chains; Top right: empirical cumulative distribution function (ECFD) for 20 chains; Bottom left: density distribution for posterior samples from 20 chains; Bottom right: lag-k autocorrelation plot, showing the correlation between each posterior sample and the sample k steps before. [file peerj-07-8100-s008.pdf]

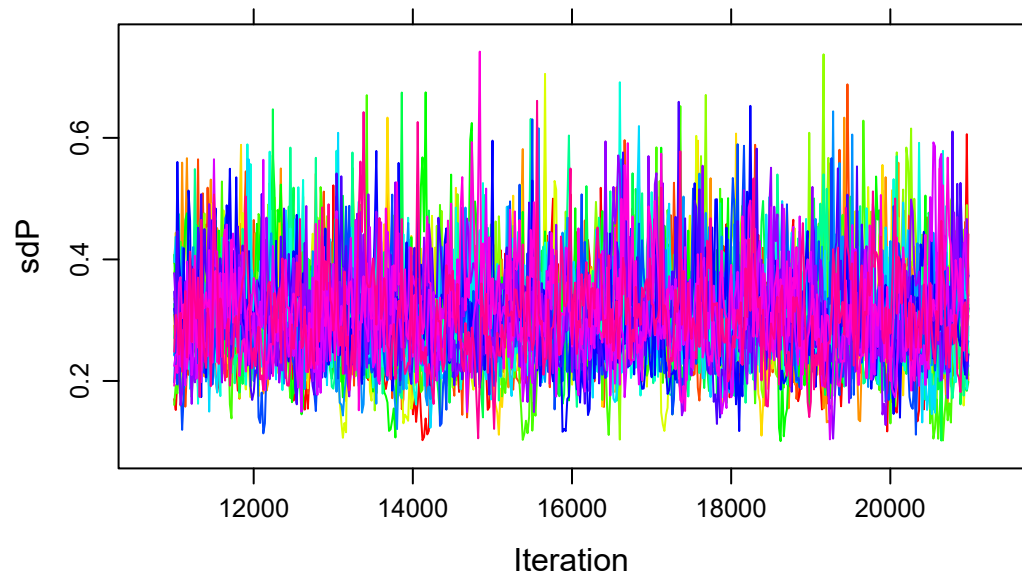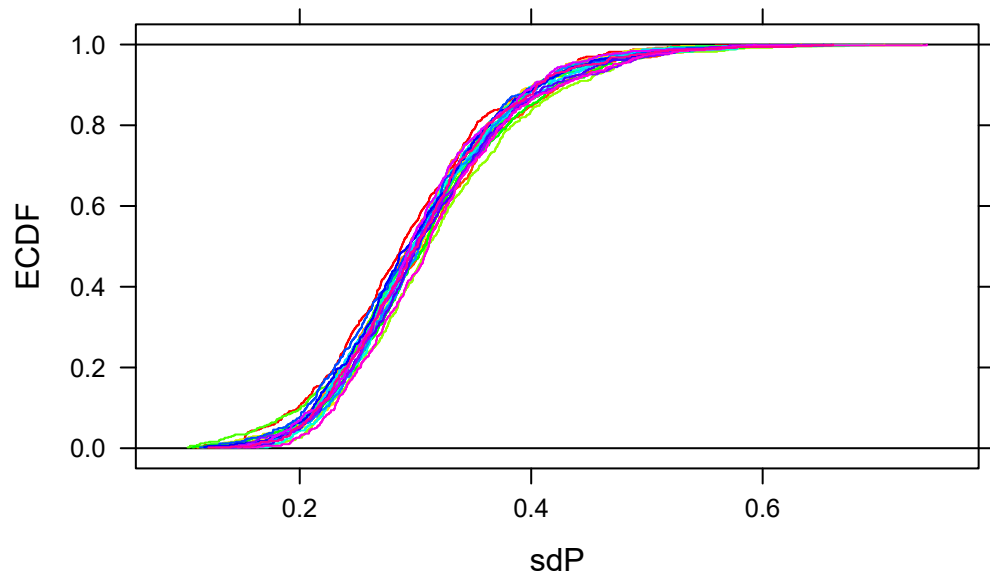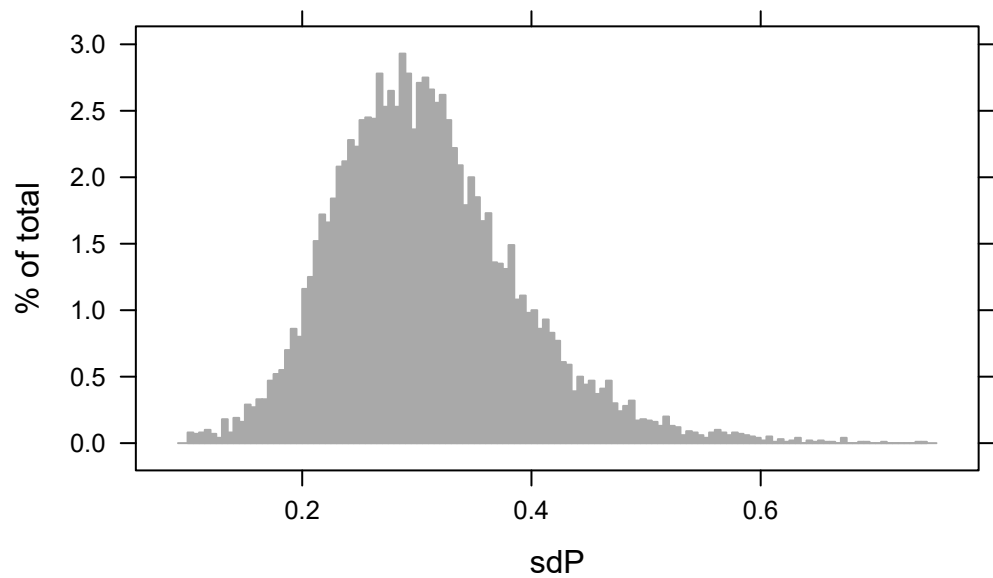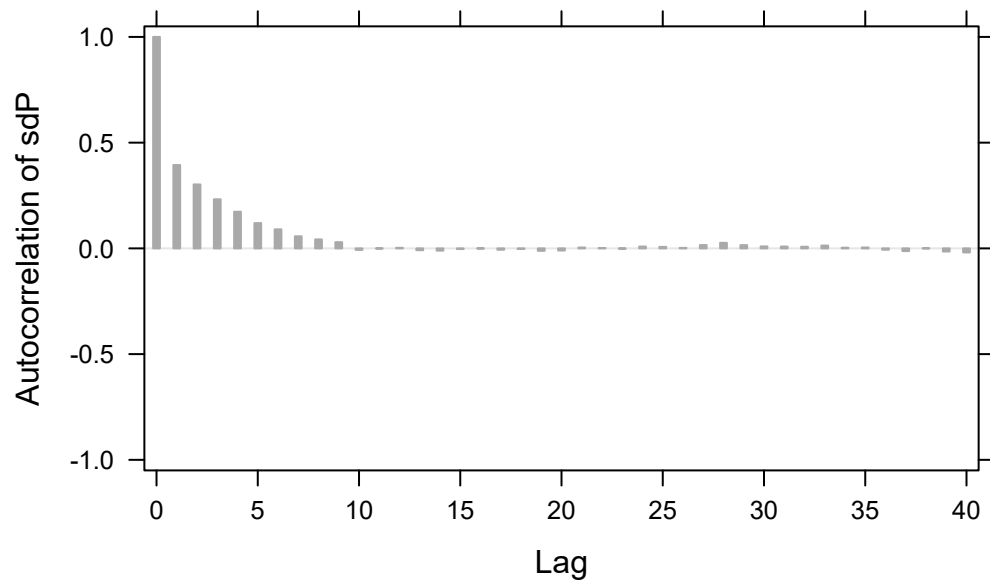

Supplement: Supplemental Information 9 — Scatterplot showing results of posterior predictive check (PPC). Posterior samples of a discrepancy statistic (summed deviance of Pearson residuals) that were generated from replicated data (y-axis) are plotted against equivalent samples generated from observed data (x-axis). Adherence of data cloud to a 1-1 line indicates good model fit, and the Bayesian P-value is the proportion of points above the 1:1 line (thus a well-fitting model should have a Bayesian P-value close to 0.5). [file peerj-07-8100-s009.pdf]

Model Sensitivity Analysis: Responses to Parameter Perturbations (-10%)

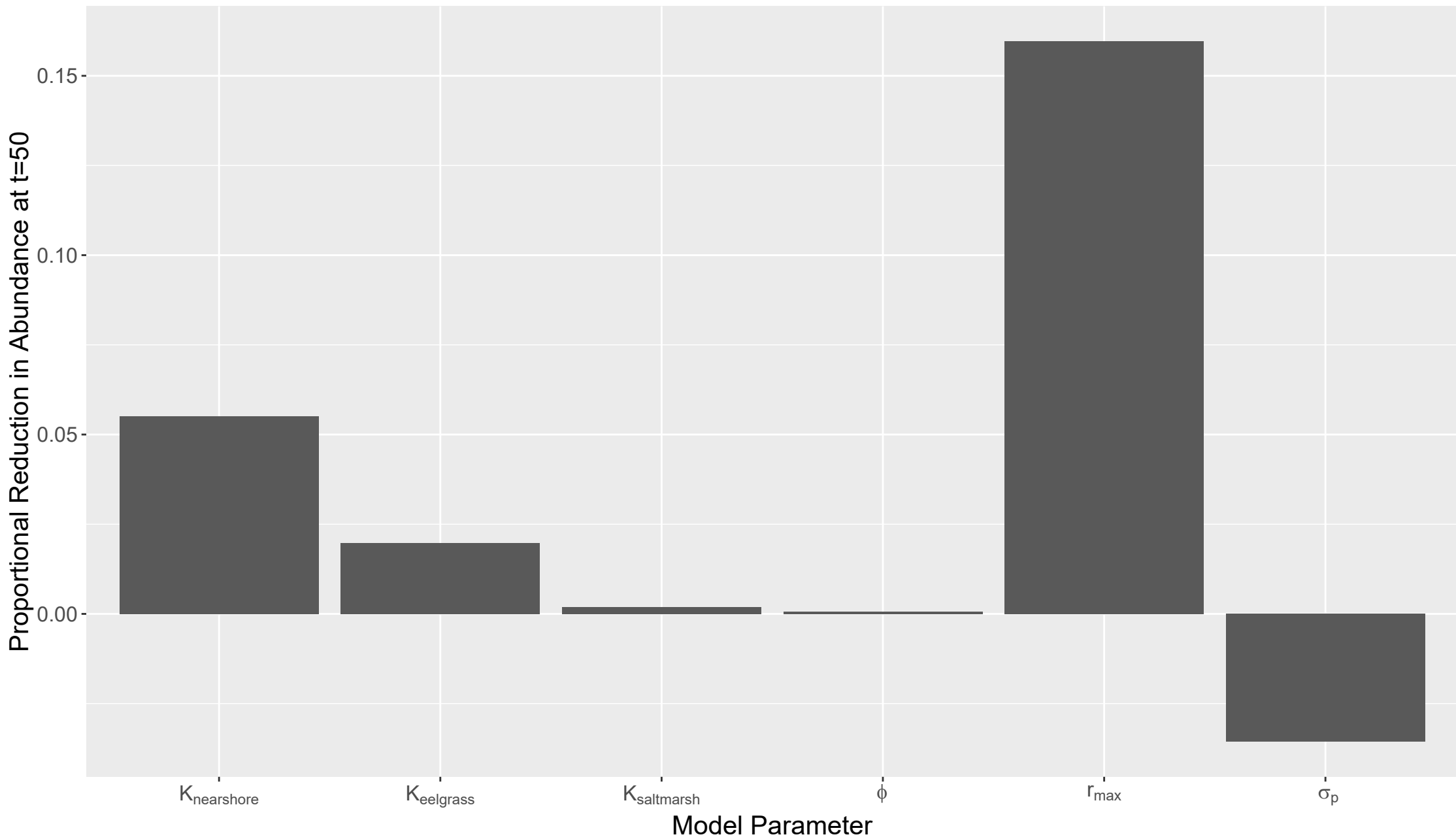

Supplement: Supplemental Information 10 — Summary of a sensitivity analysis conducted for the simulation model projecting potential growth of a sea otter population in San Francisco Bay. Column heights represent the magnitude of response in model projections (measured as the proportional reduction in N’50, the estimated abundance after 50 years) toperturbations of model parameters (parameters were individually decreased by 10%). See text for description and interpretation of model parameters. A 10% decrease in parameter “r” (maximum rate of growth) resulted in the largest proportional reduction in N’50. Note that the negative response showed for sp indicates that a reduction in this parameter lead to a proportional increase in expected N’50, rather than a reduction. [file peerj-07-8100-s010.pdf]
